# Supplementary material for: Complexome profiling of the Chlamydomonas psb28 mutant reveals TEF5 as an early PSII assembly factor
Source: Plant Cell. 2025 Mar 17;37(6):koaf055. doi: 10.1093/plcell/koaf055 (PMC12164590; doi:10.1093/plcell/koaf055)
Supplement: koaf055_Supplementary_Data [file koaf055_supplementary_data.zip › psb28_interactive.zip!/plots/intensity/qualityPlots/peptides/TPP1_Cre07.g344350.t1.2.html]

### Cre07.g344350.t1.2

Trivial name: TPP1  
SeqCoverage [median]: 25.90  
Mass: 31.03 kDa  
  
 

| Sequence | MissedCleavage | Mass [Da] | wtIntensity | muIntensity | Score |
| --- | --- | --- | --- | --- | --- |
| AGGLDDYTGVLAASTGPQQGTTAK | 0 | 2278.11 | 2534.05 | 16961.50 | 88.09 |
| FIREPVPGDVIIFHPPK | 1 | 1960.09 | 0.00 | 5393.68 | 49.56 |
| RVVAVEGDTIEVR | 1 | 1441.79 | 747.35 | 3101.26 | 123.28 |
| SEPFIAESPLYEMPR | 0 | 1764.84 | 5237.79 | 18926.85 | 176.42 |
| SFVAEPR | 0 | 804.41 | 0.00 | 2679.39 | 72.79 |
| VVAVEGDTIEVR | 0 | 1285.69 | 10753.27 | 35424.86 | 141.29 |
|  |  |  | 19272.5 | 82487.5 |  |
